# Towards a ‘smart’ cost–benefit tool: using machine learning to predict the costs of criminal justice policy interventions

**DOI:** 10.1186/s40163-018-0086-4

**Published:** 2018-10-12

**Authors:** Matthew Manning, Gabriel T. W. Wong, Timothy Graham, Thilina Ranbaduge, Peter Christen, Kerry Taylor, Richard Wortley, Toni Makkai, Pierre Skorich

**Affiliations:** 1 ANU Centre for Social Research and Methods, Australian National University, Canberra, Australia; 2 Research School of Computer Science, Australian National University, Canberra, Australia; 3 Jill Dando Institute of Security and Crime Science, University College London, London, UK; 4 Australian Public Service, Canberra, Australia

**Keywords:** Cost–benefit analysis, Machine learning, Cost–benefit tools, Data science

## Abstract

**Background:**

The Manning Cost–Benefit Tool (MCBT) was developed to assist criminal justice policymakers, policing organisations and crime prevention practitioners to assess the benefits of different interventions for reducing crime and to select those strategies that represent the greatest economic return on investment.

**Discussion:**

A challenge with the MCBT and other cost–benefit tools is that users need to input, manually, a considerable amount of point-in-time data, a process that is time consuming, relies on subjective expert opinion, and introduces the potential for data-input error. In this paper, we present and discuss a conceptual model for a ‘smart’ MCBT that utilises machine learning techniques.

**Summary:**

We argue that the Smart MCBT outlined in this paper will overcome the shortcomings of existing cost–benefit tools. It does this by reintegrating individual cost–benefit analysis (CBA) projects using a database system that securely stores and de-identifies project data, and redeploys it using a range of machine learning and data science techniques. In addition, the question of what works is respecified by the Smart MCBT tool as a data science pipeline, which serves to enhance CBA and reconfigure the policy making process in the paradigm of open data and data analytics.

## Background

In recent years, cost–benefit analysis tools, such as the Manning Cost–Benefit Tool (MCBT)^1^ (Manning et al. 2016) and the Washington State Institute of Public Policy’s (WSIPP) Benefit–Cost Tool (Aos and Drake 2010), have been developed to assist criminal justice policymakers, policing organisations and crime prevention practitioners to assess the benefits of different interventions for reducing crime and to select those strategies that represent the greatest economic return on investment. A framework for evaluating the effectiveness of crime prevention policies or alternatives, including economic benefits, has been proposed recently by Johnson et al. (2015). The authors describe “…the need for, and the development of, a coding system to distil the quality and coverage of systematic reviews of the evidence relating to crime prevention interventions” (p. 459). EMMIE, the coding scheme developed by the authors, assesses the probity, coverage and utility of available empirical evidence. Five dimensions are identified including: (1) effect of intervention; (2) the identification of the causal mechanism(s) through which interventions are intended to work; (3) the factors that moderate their impact; (4) the articulation of practical **i**mplementation issues; and (5) the **e**conomic costs of intervention.

The What Works Centre for Crime Reduction (College of Policing UK 2017), which adopts the EMMIE rating scale, shows that the available economic evidence from prevention studies is either non existent or inadequate across a wide range of crime intervention types. One possible reason for the lack of economic evidence is the difficulty in using current economic tools where users need to input, manually, a considerable amount of point-in-time data in order to provide a range of economic estimates to inform decisions about the allocation of finite government resources. This process is time-consuming and also introduces potential input errors as human users become fatigued and/or complacent. Further, users are faced with the complication of estimating projected costs in different jurisdictions and environments and of relying on information which may be out of date. These factors are important when estimating the costs associated with interventions across different locations involving potential contextual variation. Currently, only the MCBT is capable of estimating such costs across environments, but the tool is limited to operating on expert opinion based on experience and subjective judgement.

In this paper, we examine how machine learning and data science techniques can be applied to the MCBT—to produce a ‘Smart MCBT’—in order to help overcome the above mentioned obstacles, as well as to produce new insights and analyses of economic data. The Smart MCBT is not limited to criminal justice applications and can be easily adapted for evaluation in any field (e.g. health, environment, engineering, etc.) and at any level (e.g. small to large business, government, etc.).

The paper begins by outlining the concept of economic analysis as it applies to resource allocation and discusses existing tools for undertaking this task. A detailed description of the MCBT follows, discussing its benefits and limitations. Next, we provide a description of the proposed architecture of the Smart MCBT and an application of key machine learning techniques. Finally, we discuss the implications of the Smart MCBT and its future development.

## Economic analysis and the allocation of resources

Crime prevention requires decisions regarding the use of inputs and how these inputs translate into the most effective and sustainable outcomes. The choice of intervention will be influenced by factors including, but not limited to, budget, the crime problem being addressed, the environment in which the crime is taking place, social and ethical considerations, and judgements about the relative effectiveness of alternative interventions.

The decision about what is the most efficient allocation of resources for crime prevention is not an easy one because: (1) budgets are limited and attempting to anticipate the costs associated with an intervention is difficult, typically based on previous implementations in other jurisdictions and locations; and (2) there is an opportunity cost where resources used for one application are at the expense of other possible applications. To make this decision effectively, empirical evidence regarding the costs and benefits of alternative actions needs to be undertaken.

Policy-makers are presented with three critical questions the answers to which inform decisions about the allocation of resources to a given course of action: what does the intervention cost? how effective is the intervention? and, what are the externalities (i.e. positive and negative side-effects)? Economic analysis (EA) provides answers to these empirical questions. EA is designed to provide a rational basis for the allocation of scarce public resources that leads to a set of socially desirable outcomes while minimising undesirable economic and social impacts (Boardman et al. 2006; Manning et al. 2016a, b). Thus, EA promotes economic efficiency and good fiscal management. Specifically, results generated from EA are used to assess available options with the aim of identifying those interventions (or combinations of) that provide the greatest economic return on investment. In addition, results from EA can be used by policymakers to gauge the economic implications of existing interventions and provide insight into the cost of inputs required to undertake an intervention in another location (Manning 2004, 2008). The five main methods of EA are: cost-feasibility analysis (CFA), cost-savings analysis (CSA), cost-effectiveness analysis (CEA), cost-utility analysis (CUA) and cost–benefit analysis (CBA). Table 1 provides a summary of the main methods and their respective advantages and disadvantages. A detailed comparison of these methods can be found in Manning et al. (2016a, b).

**Table 1:** Common EA techniques. Adapted from Manning et al. (2016)

Type of analysis	Measure of cost/inputs	Measure of outcomes	Strengths	Weaknesses	Analytical questions	Example/s
Cost-feasibility (CFA)	Monetary value of resources	N/A	Permits alternatives that are not feasible to be immediately ruled out before evaluating outcomes	Cannot judge overall worth of a project because it does not incorporate outcome measures	Can a single alternative be carried out within budget?	(Manning 2004; Manning et al. 2006)
Cost-effectiveness (CEA)	Monetary value of resources used during implementation	Units of effectiveness (e.g. crimes prevented or treatments delivered)	Easy to incorporate standard evaluations of effectiveness Good for comparing the cost of delivery per unit of treatment across interventions Good for alternatives with a small number of objectives	Hard to interpret if there are multiple measures of effectiveness Only useful for comparing two or more alternatives	Which alternative yields a given level of effectiveness for the lowest cost (or highest level of effectiveness for a given cost)?	(Cowell et al. 2004; McCollister et al. 2004)
Cost-savings	Monetary value of resources used during implementation	Monetary savings resulting from impact of intervention	Good for assessing the savings generated to stakeholders	Difficult to place monetary values on salient life benefits	What are the estimated savings generated from the intervention?	(Manning et al. 2006)
Cost–benefit (CBA)	Monetary value of resources used during implementation	Monetary value of benefits	Can judge absolute worth of a project Can compare CB results across a variety of projects	Difficult to place monetary values on salient life benefits	Which alternative yields a given level of benefits for the lowest cost (or the highest level of benefits for a given cost)? Are the net benefits greater than the net costs?	(Yeh 2010)
Cost-Utility (CUA)	Monetary value of resources used during implementation	Units of utility	Incorporates individual preferences for units of effectiveness Incorporates multiple measures of effectiveness onto a single measure of utility Promotes stakeholder participation in stakeholder decision making	Difficult to derived consistent and accurate measure of individual preferences Cannot accurately judge overall worth of a single alternative Only useful for comparing two or more alternatives	Which alternative yields a given level of utility for the lowest cost (or the highest level of utility for a given cost)?	(Dijkgraaf et al. 2005; Dolan and Peasgood 2007)

A number of guidelines and tools have been developed to assist in deriving reliable and comparable EA estimates. Examples of guidelines include: the Regulatory Impact Analysis Inventory (Organisation for Economic Co-Operation and Development 2004); the Green Book United Kingdom (HM Treasury 2003); the Cost–Benefit Analysis Guide, Australia (Department of the Prime Minister and Cabinet 2016); and in the United States, the Regulatory Impact Analysis (Circular A-4) (The Office of Management and Budget 2003). Examples of tools include: WSIPP’s Benefit–Cost Tool (Aos and Drake 2010), which allows the state to predict the impact of policy options and to calculate the net present values, cost–benefit ratios and projected returns-on-investment from prevention and intervention programmes and policies; and the MCBT (Manning et al. 2016), which operates in Microsoft Excel. A full summary of the above guidelines and tools is provided in Manning et al. (2016a, b). In the following section a detailed description of the MCBT is presented.

## The Manning Cost–Benefit Tool (MCBT)

The MCBT, as well as producing similar outputs to those produced by WSIPP’s tool, employs traditional costing techniques (see for example HM Treasury *Green Book* 2003). The MCBT allows for the calculation of cost-savings, cost-effectiveness, cost-feasibility and cost–benefit ratios as well as net returns on investment. Importantly, the tool disaggregates results by stakeholder. In addition, the MCBT allows for the comparison of average annual expenditure before and after the introduction of the intervention. Uniquely, MCBT adopts a combination of methods (e.g. multi-criteria analysis) for calculating the costs of an intervention in the absence of reliable accounting data (Manning et al. 2016a, b).

While existing EA tools and guidelines are helpful for estimating costs of an existing intervention, only the MCBT provides potential costs of an intervention when implemented in a different location/jurisdiction, particularly where obvious contextual variation exists. The importance of this cannot be overstated because most responses to crime require interventions that have been employed elsewhere. Decision makers need to assess how much of an input to use in a different location or jurisdiction when many of the salient variables that affect outcomes are different.

**Fig. 1:**
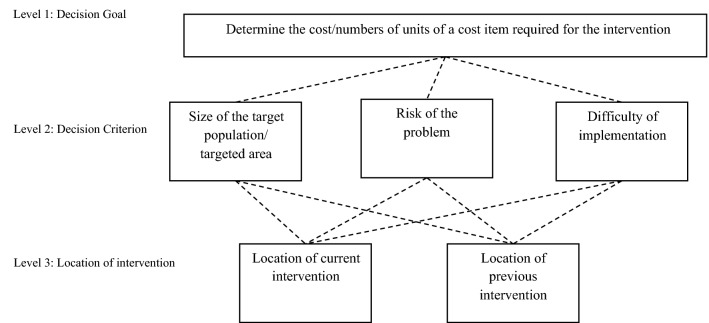
Decision mode hierarchy in the existing MCBT

The developers of the MCBT highlight three context-specific factors that are typically associated with the implementation of an intervention and which are likely to affect costs: (1) the size of the population/targeted area; (2) the perceived risk of the problem; and (3) the perceived difficulty of implementation. According to Manning et al. (2016a, b), the cost of implementing a similar intervention in different locations may depend on variations in one or more of these three variables. Figure 1 illustrates the decision hierarchical structure of the MCBT when an existing intervention is to be implemented in a different location. The decision goal (i.e. determine the cost/numbers of units of a cost item required for the intervention) is represented on level 1. Level 2 highlights the possible contextual variables that need to be considered and assessed with regards to their relative influence on the overall cost. Level 3 compares the variation between two locations with respect to the criteria represented on level 2. The above assessment undertaken on levels 2 and 3 using analogous estimation^2^ and expert judgement provides weights with regards to how much each cost item should be adjusted to reflect the contextual differences across locations. For a full explanation of the method refer to Manning et al. (2016a, b).

While the use of analogous estimation and expert judgement in the MCBT allows evaluators to estimate costs (i.e. direct, indirect and intangible) based on variations in given contextual factors, challenges faced by evaluators include: (1) the constraint in incorporating additional variables into the estimation because of the limitations of Microsoft Excel (the underlying modelling tool); (2) the use of expert judgement which may lead to biased results (likely to be more conservative results as the tool advises the application of an optimism bias adjustment when using expert judgement); (3) the restriction on the use of multiple data sources; and (4) the lack of insights which could be gained from the data over time.

**Fig. 2:**
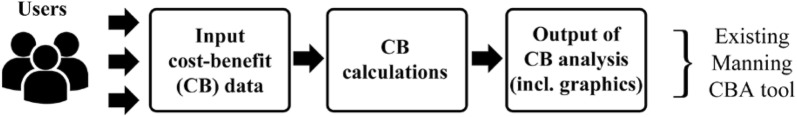
The existing MCBA tool data flow

Figure 2 provides an overview of the ‘data flow’ of the MCBT. As shown in the figure, the MCBT comprises three sequential modules that combine to measure the costs and benefits of a crime intervention project. Each of these modules is described in the following sections.

### Module 1: Input cost–benefit data

The MCBT currently requires that the evaluator provides all necessary data for the estimation of annual and total costs/benefits through direct data input or indirect calculation with the use of quantity and per item values. For example, consider the use of ‘gated communities’ as a crime prevention strategy. Blakely and Snyder (1998) define gated communities as:

…residential areas with restricted access that makes normally public spaces private. Access is controlled by physical barriers, walled or fenced perimeters, and gated or guarded entrances… They represent a phenomenon different from apartment or condominium buildings with security systems or doormen. There, a doorman precludes public access only to a lobby or hallways - the private space within a building. Gated communities preclude public access to roads, sidewalks, parks, open space, and playgrounds - all resources that in earlier eras would have been open and accessible to all citizens of a locality (p. 53).

Gated communities are designed primarily to deter crime, but they also act as barriers, which may enhance the freedom and wellbeing of residents by eliminating daily annoyances (e.g. canvassers) and malicious behaviours (e.g. mischievous teenagers) (Blakely and Snyder 1998).

In order to model the costs and benefits associated with gating a community, data could be drawn from several sources (e.g. independently audited cost data, formal service delivery contract costs, security management costs, costs developed from ready reckoners and uncorroborated expert judgement) and in different forms (market values such as salary and equipment costs or non-market values such as sense of security) to define the scope (i.e. degree of inclusion of costs and benefits of relevant stakeholders) and depth (i.e. estimation of tangible and intangible costs/benefits) of the analysis (see Manning et al. 2016a, b).

### Module 2: Cost–benefit calculations

In the MCBT, costs and benefits are calculated taking into account the economic assumptions (i.e. inflation and discount rates), confidence intervals (i.e. worst and best-case scenario), optimism bias correction, percentage of total cost borne and spent each year and attributable fraction (i.e. percentage or proportion of costs attributed to the intervention of concern).

Typically, when the cost of an item is entered, this cost will be upwardly corrected, based on the identified optimism bias,^3^ to reduce the likelihood of cost underestimation due to less reliable inputs because of the source and age of data (HM Treasury 2014). Likewise, benefits are downwardly adjusted to avoid overestimation of benefits. The adjusted costs/benefits based on optimism bias correction are used for the analysis. Evaluators specify how much of the total cost/benefit of an item were spent/gained each year. This adjustment allows the MCBT to calculate the annual cost/benefit, taking into account the worst-case (i.e. the intervention costs more money; 1+ margin of error), the best-case (i.e. costs less money; 1− margin of error) and the expected case (i.e. the average), thus providing a confidence interval (the default is 95%). After deriving the overall costs for each year (over the lifespan of the intervention), an attributable fraction^4^ may be applied to yield program-specific costs. With the estimation of program-specific costs for each year, the costs are then adjusted for inflation and discount rate.

This procedure is also applied to the benefits (avoided costs) of the intervention, where the evaluator provides cost data for each selected crime type and information for the calculation of net savings and benefits. The cost per crime avoided is calculated by dividing the intervention costs by the unit of effect (e.g. number of crimes avoided). The cost-effectiveness ratio is calculated by dividing the annual costs by avoided criminal incidents. The cost–benefit ratio is calculated by dividing the economic benefits with the intervention costs. Separate calculations are also performed for each bearer/stakeholder. It should be noted that there are also potential benefits beyond the avoided costs of crime, such as where an offender becomes employed and no longer receives government benefits. The benefit here is not the reduction in welfare payments, as this will be considered a transfer payment,^5^ but rather it is the additional income tax received as a result of the employment and the contribution this work makes to the overall economy. Such benefits are captured as ‘additional benefits’ in the MCBT.

### Module 3: Output of cost–benefit analysis

All of the outputs in the existing MCBT are presented in tables (i.e. net costs, net benefits, cost–benefit ratio and net benefits by bearers) and plots. Outputs are listed in Table 2 below. In summary, MCBT allows for CFA (i.e. comparing the overall costs of the project against the budget), CSA (i.e. comparing the costs of the project against the savings generated from avoided crimes), CEA (i.e. comparing the costs of the project against the number of units of output such as the number of crimes prevented) and CBA (i.e. comparing the costs of the project against the overall benefits-avoided crimes and other benefits such as enhanced safety), as discussed in Table 1.

**Table 2:** Outputs of the MCBT

Excel tab	Description
Cost section	Calculation of overall expenditure of one or more intervention programs
Total costs	A display of the total costs after the implementation of a program, including both best and worst case scenarios, and compares costs of the intervention with the status quo
Costs with economics	A display of the costs with and without inflation and discount rate for each year
Costs to bearers	A display of the costs for each bearer, including the costs of each year of intervention, total costs of the intervention and the average annual costs
Benefit section	Calculation of financial benefit of a program
Cost-effectiveness	A display of the cost-effectiveness outcomes including the cost-effectiveness ratio
Cost–benefit	A display of the cost–benefit outcomes including the cost–benefit ratio and net benefit
Benefits with economics	A display of the benefits with and without inflation and discount rate for each year
Savings and benefits to bearers	A display of the benefits for each bearer/recipient, including the benefits by year of intervention, total benefits of the intervention and the average annual benefits

## The development of a smart MCBT

As identified in the introduction, there are a number of limitations with the MCBT. These include: (1) users manually inputting a sizeable quantity of point-in-time data; (2) the potential of input errors to arise as users become fatigued and/or complacent; and (3) the difficulty in estimating projected costs in different jurisdictions and environments (i.e. contextual variation) and relying on information which may be out of date. In view of these limitations we propose the development of a Smart MCBT, which incorporates data science and machine learning techniques. Here, we argue that much can be learned through interdisciplinary collaboration. In this paper, our focus is on the amalgamation of criminology, economics and computer science with respect to the advancement of the MCBT. An enhanced capacity to assess programme costs and benefits is particularly important in the area of crime and justice since few interventions—as highlighted by the Crime Reduction Toolkit (College of Policing UK, 2017)—are accompanied by EA, and when they are the EA tends to be of limited quality. However, it should be noted that the Smart MCBT is not limited to criminal justice applications alone and can be easily adapted for evaluation in any field (e.g. health, environment, engineering etc.) and at any level (e.g. small to large business, government etc.).

**Fig. 3:**
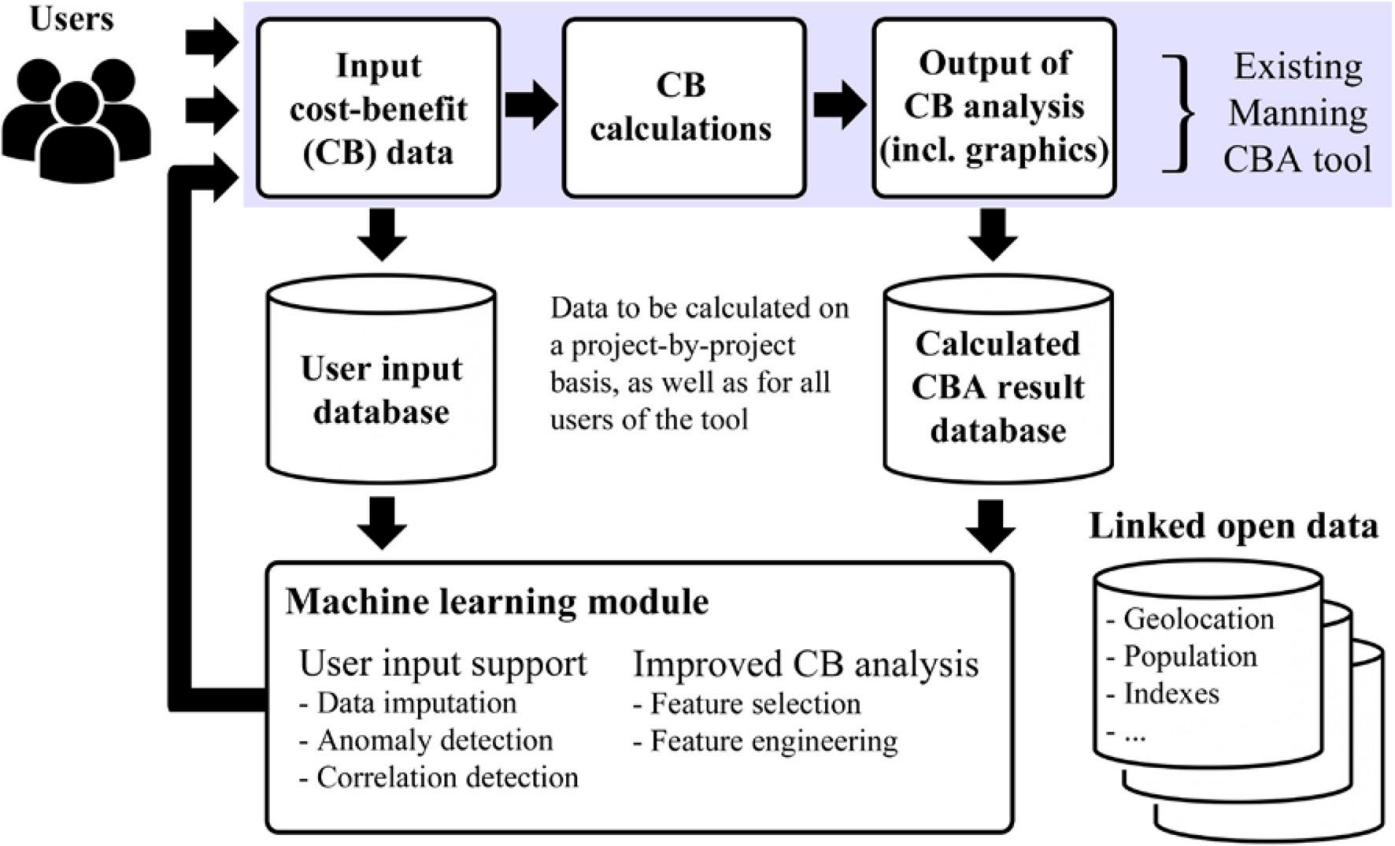
System data flow for Smart MCBT

The proposed Smart MCBT is illustrated in Fig. 3, where the boxes indicate a system process or database, and the arrows indicate the direction in which data ‘flows’ from one component to the next. As previously discussed, the existing MCBT has three main components (shown in Fig. 2 and highlighted in blue in Fig. 3). We will discuss the four newly proposed components (Modules 4–7) in the sub-sections that follow.

### Module 4: User input database

For the existing MCBT, input data entered by the user are only stored locally on a per-project basis. This provides the first obstacle to building a tool that draws upon and exploits machine learning (ML) techniques to improve the system. Therefore, in the ‘User Input Database’ component, all data entered by users into the MCBT is stored as one set of records per project. The Smart MCBT will overcome this limitation by storing input data on a shared server, providing a single data resource for the ML Module.

Returning to our earlier example of gated communities, we propose a list of some (but not all) relevant variables that would be required in determining the cost associated with such an intervention. In Table 3 we present four predictors (independent variables or IVs) that may affect the cost associated with gating a vulnerable community (dependent variable or DV). Drawing on data from multiple inputs, evaluators, for example, may have data on the number of burglaries that have occurred in each community under review. The Smart MCBT will gather and store these data for subsequent future analyses and current estimations.

**Table 3:** Examples of variables—cost of gating a community to prevent crime, annoyances and malicious behaviour

Variable	Description	Type
Costs associated with restricting access to only local residents and their visitors to reduce opportunities for crime, eliminate daily annoyances and malicious behaviours	Cost of gates the community	DV
Size of area	The geogrpahical properties of the area to be gated	IV
Access points	The number of access points required	IV
Guard box	The proportion of access points where a guard box is required	IV
Number of burglaries	Burglaries that occurred in properties with clearly identifiable facilitating opportunities	IV

### Module 5: Calculated CBA database

Where Module 4 provides a database to store the user input data, the ‘Calculated Cost–Benefit Analysis Database’ (herein CCBA-DB) provides a database to store the calculated results *after* analysis. Storing the calculated results is crucial because it provides the ability to model the relationships between all input-relevant cost and benefit data (Module 1) and the output of CBA. With the existing MCBT, the knowledge generated from a CBA of a particular intervention is not able to leverage the results of previous analyses, even when the cost and benefit data (the existing tool input and output) are similar or the same, subject to contextual variation. Therefore, the database in the CCBA-DB component of the workflow diagram stores the benefits (that could also be weighted using a harm index^6^) and analysis data, enabling the system to map the relations between input and output, and exploit this to learn and improve CBA over time. Returning again to the hypothetical example, Module 5, similar to Module 4, will store these outcome data (e.g. cost of installation of gates).

### Module 6: Machine learning module

The sixth module comprises a range of data science techniques and ML algorithms that contribute the main improvements of Smart MCBT. Broadly speaking, ML is the field of computer science that studies how to enable computers to act and learn without being explicitly programmed. Formally, “… a computer program [through ML] is said to learn from experience E with respect to some class of tasks T and performance measure P if its performance at tasks in T, as measured by P, improves with experience E” (Mitchell 1997, p. 2). ML is divided into two main areas, ‘supervised’ and ‘unsupervised’ ML (Hastie et al. 2009).

The Smart MCBT uses supervised and unsupervised techniques. Supervised ML involves learning from labelled data, such that the task involves estimating a model that optimally maps input variable(s) to output variable(s). It is therefore able to correctly predict the label (classification) and/or predict values (regression) for new observations where the actual ‘ground truth’ labels or values are known but not provided to the algorithm during the training process. For example, a machine can learn to identify whether a photo includes a cat (or not) if it is provided a set of photos (represented in a suitable digital format) and labels for each photo (denoting whether the photo is of a cat or not). If we ‘train’ the model on 80% of the labelled photo data, we can then use the remaining 20% to ‘test’ how well the model does at correctly predicting whether a cat is featured in each photo. The model is said to have learned from the data in a supervised fashion because it needs to be instructed or ‘trained’ on photos that have a known ground-truth label. On the other hand, unsupervised ML describes a broad range of techniques that aim to model or infer hidden structure from unlabelled data. For example, suppose we have a collection of photos that are portrait shots of different kinds of animals; however, we do not have labels to describe which animals are in each photo. An unsupervised clustering approach could be used in this scenario to discover implicit groupings within the photo data, such that photos are grouped into clusters based on their similarities and differences. The resulting clusters would ideally contain photos of the same kinds of animals (e.g. cats in cluster 1; elephants in cluster 2, and so on).

The integration of ML techniques in the Smart MCBT is designed to achieve two main goals, namely: (a) to provide input support to the user by predicting missing values, identifying potentially erroneous values, and making suggestions about relevant contextual factors; and (b) to improve the analytical capabilities of CBA by usefully reducing the number and types of variables to minimize user effort (e.g. time-consuming data entry) and develop better estimates (e.g. cost savings; crimes avoided), based on what the system learns from previous similar projects. We now turn our attention to each of these techniques to elaborate how they are deployed in the design of the Smart MCBT.

#### User input support

As shown in Fig. 3, Module 6, conceptualised as ‘User Input Support’, comprises three tasks—data imputation, anomaly detection, and correlation detection—which provide input support and recommendations for the user. The first task, data imputation, is to find the best values for uncertain or unknown input values based on other interventions of a similar nature. Earlier, we discussed the issue of undertaking CBA when contextual variation exists. We can illustrate the process by drawing again on the gated community example, keeping in mind the relationship between the DV and IVs as shown in Table 3. In this example, we include other environmental crime control processes to augment the use of gating a community to reduce property crimes (e.g. the use of guard posts in a gated community). If a manager of an environment (e.g. complex manager of a gated community) imposes new procedures regarding unsupervised entry into the complex, the Smart MCBT should be able to perform two tasks: (1) suggest to the user what kinds of contextual variation factors are relevant (e.g. number of possible entry points into a complex based on geographical data); and (2) estimate or impute the best values for uncertain data input. For example, a user might have available data on most of the IVs listed in Table 3, but a paucity of data on number of guard boxes that may be required given the size and layout of the complex. In this situation, the tool would be able to estimate and suggest the best values to the user by calculating appropriate values based on similar previous interventions with similar input patterns. To achieve user input support in this context, we propose to implement and evaluate a combination of multivariate imputation and ML techniques for missing data.

Multivariate imputation (MI) is a method for estimating incomplete data based on predictions derived from observations in the dataset (Rubin 1996; Allison 2000). This technique has been recently developed using an approach based on chained equations, or Multiple Imputation by Chained Equations (MICE) (van Buuren and Groothuis-Oudshoorn 2011). However, as Allison (2000) argues, one of the key assumptions of MI is that the data are missing at random (MAR), such that the probability of missing values in a variable Y depend only on the information contained in other variables and not Y itself. This issue is compounded when missing values exceed a certain percentage of observations, whereby MAR can no longer be reasonably assumed. Indeed, violations of the MAR can be expected in many real-world cases (Schafer and Graham 2002), although fortunately such violations have not been found to seriously bias parameter estimates for missing data (Collins et al. 2001). Nevertheless, to further address issues that can arise due to the assumptions and statistical properties of standard techniques for imputation of missing data, we propose to supplement and potentially enhance these with newer machine learning (ML) approaches.

Studies suggest that ML approaches to missing data perform on par with, and increasingly outperform, MI methods such as MICE. Richman et al. (2007) compared supervised ML algorithms with standard imputation techniques and found that support vector machines (SVM) and neural networks had the lowest error rate, and are particularly suited to scenarios where a large percentage of missing data is present. Similarly, Schmitt et al. (2015) found that data imputation through bayesian principal component analysis and fuzzy K-means outperform more standard and popular methods, notably lower error rates than multiple imputation using the MICE approach. Recent developments in nonparametric missing value imputation using a random forest approach, or MissForest (Stekhoven and Bühlmann 2012), have also been found to outperform standard methods, including MICE (Waljee et al. 2013), although the computational cost is considerably higher. As MI and ML have different strengths and limitations we propose that the Smart MCBT integrate both these methods of dealing with missing data.

The second task that the ‘User Input Support’ sub-component addresses—anomoly detection—involves identifying abnormal input values (see Christen et al. 2016) that could be erroneous, whether random errors (e.g. a mis-typed number) or systematic errors (e.g. incorrectly entering the data from variable X into the fields for variable Y). The system would calculate this by comparing the data from other similar interventions and using anomaly and outlier detection methods to identify values in the current intervention input data that do not conform to the expected values or patterns in the input data of other similar interventions. Proposed algorithms to identify such (potential) errors include density-based techniques such as k-NN (Altman 1992) and the local outlier factor (Breunig et al. 2000), as well as ensemble methods for outlier detection in multivariate data (Kannan and Manoj 2015). By identifying potential erroneous input values, the system is able to notify the user at an early stage, and moreover gains higher accuracy over time as more interventions (and therefore more data examples) become available.

In terms of providing ‘smart’ user support for CBA of a given intervention across multiple jurisdictions, outlier detection will be useful for identifying and predicting contextual variation between similar intervention types. Certain input variables or sets of variables may conform to known distributions or estimable functions. If the system is able to map these patterns, then it can detect whether the input values for a similar intervention are deviating in an unusual way, whether for individual input values, individual variables, or multiple variables.

The third task of User Input Support, correlation detection, can be performed on the input data to identify variables that are collinear, or more generally to identify multicollinearity in the input data. This is useful feedback to the user because it will reveal whether, and to what extent, they are entering variables that are basically highly inter-correlated and contain similar information, or are even the same variable and simply duplicated. Smart MCBT becomes even more useful as these kinds of patterns are identified across multiple similar interventions, which might suggest a re-evaluation of the input data or costs associated with the intervention.

Through this process, whereby contextual variation factors associated with common intervention strategies will be identified, and assisted with expert knowledge, the Smart MCBT can build up an ontology of contextual variation factors that apply to common problems or intervention strategies. Such an ontology, presented in the standard Web Ontology Language (W3C 2012), will act as both a knowledge base and a taxonomic *lingua franca* for interventions and contextual variation factors. It can then be used to improve the relevance of the tool’s suggestions for contextual factors. The ontology, built partly through use of the tool and representing an expert conceptual model of the problem domain, can then be re-used to enrich pattern analysis and feature discovery as elaborated below. Furthermore, it can also be used for leveraging external *linked open data* resources, also elaborated below.

#### Improved CB analysis

The second sub-component of the ML Module 6 involves using ML and data science techniques to improve the analytical capabilities of the existing MCBT. As shown in Fig. 3, this sub-component receives data from the *input* and *output* of the CBA, for example, the number of crimes avoided in a particular jurisdiction. Collecting both the input and output data provides a strong basis to deploy supervised ML. For example, using our gated community approach, body corporate resources could be spent on a range of alternatives to prevent burglary, but the question is—what alternative or combination of alternatives should they focus on or how can they maximise the use of resources to produce the greatest reduction in crime opportunities within budget? We argue that posing this as a ML problem has significant potential to enhance the analysis and outcomes, both in terms of costs saved and benefits increased and in terms of our understanding of the complex relationships between interventions and outcomes. In other words, ML can reveal how well can we predict a set of benefits from a set of costs.

CBA can be improved through ML and data science techniques by specifying the variables using two techniques—‘feature selection’ (Hastie et al. 2009) and ‘feature engineering’ (Zheng 2016). Feature selection is concerned with selecting a *subset* of variables or features that are most relevant for constructing the ML model. As Guyon and Elisseeff (2003) argue, “The objective of variable selection is three-fold: improving the prediction performance of the predictors, providing faster and more cost-effective predictors, and providing a better understanding of the underlying process that generated the data” (p. 1157). In this way, feature selection can result in ‘knowledge discovery’ whereby previously unknown information about the data can be extracted and put to use.

An approach to feature selection is to understand a structured ontology of crimes, interventions and contextual factors, representing both expert knowledge and structural relationships amongst features. This ‘structured ontology’ provides a common language to describe the types, properties, and relationships of all the variables for CBA projects. Research has developed highly effective semantic ML techniques that can leverage such an ontology to discover patterns in data (Ratcliffe and Taylor 2014, 2017). Using a search process over the semantic relations expressed in the ontology, it is possible to propose compact and highly readable pattern descriptions that exploit combinations of features that are not directly present in the data. For example, let us assume that the assembled MCBT historical data sometimes include detailed descriptions of gated community designs and co-located lighting designs that have been tried in a number of independent interventions. Given suitable ontology modelling of gating designs, lighting designs, and localised daylight hours, a semantic ML technique could be expected to distinguish effective and ineffective interventions with descriptions like “*gates are made of any metallic material excluding aluminium, illuminated after dark and during dusk*”, or perhaps “*gates are above 3* *m high, coloured brightly, and all access points are guarded/or monitored electronically (e.g. CCTV)*”. This significantly enhances CBA because it ‘joins up’ similar, but otherwise siloed, projects (subject to contextual variation) by exploiting a reservoir of expert and domain knowledge embedded in the system.

Feature engineering, on the other hand, is a process that involves using domain knowledge of a particular region to generate a set of variables, or ‘features’, which ML can be applied to. Currently, feature engineering for the MCBT involves expert opinion based on experience and subjective judgement. For example, official statistics (e.g. police recorded crime statistics) may show an increase in burglaries in areas that appear vulnerable due to crime facilitating conditions (e.g. lack of capable guardians, or a lack of target hardening measures). Therefore, crime facilitating conditions and the corresponding crime opportunities would be included as a feature of the CBA data set for a given project about potential use of gating as a means of reducing crime.

### Module 7: Linked open data

The existing MCBT facilitates an analysis of user-defined cost variables (input features) and benefit variables (output features). These normally result from feature engineering processes conducted by domain experts (e.g. regulators and lawmakers), drawing on subjective experience and domain-specific training. In practice, the features are determined by the person responsible for setting up the intervention and entering data into a CBA tool. Whilst the current MCBT affords a robust and state-of-the-art analysis, it is also limited because it does not automatically take advantage of external sources of data that might be useful or relevant for the decision-making process.

Governments are increasingly making data publicly available through “Open Data” initiatives, such as Data.gov.uk (UK), Data.gov (US), and Data.gov.au (Australia). For example, Data.gov.au provides nearly 30,000 discoverable datasets, along with 6000 application programming interfaces enabled resources across multiple areas, including environment, community services, health care and more. Importantly, as open data and national statistics agencies evolve and improve, the data they provide is increasingly available in standardised formats accompanied by metadata that informs us about the nature and structure of the data objects. Of particular importance is data published as *linked open data* (Bizer et al. 2009) that relies on open Web principles for publishing, discovery and retrieval, supported by machine-processable ontologies for data description (Haller et al. 2017). We argue that it is precisely these kinds of open data and emerging standards for handling such data that could be identified and deployed within a Smart MCBT approach.

## Discussion

In this paper we have outlined and examined a proposed architecture for a Smart CBA tool that incorporates a range of ML and data science techniques. From a user and agency perspective, the Smart MCBT has the potential to speed up data entry via imputation, minimise input errors via anomaly detection and facilitate knowledge discovery through simplified predictive models reports and graphs generated from automated data analysis. From a policy and governance perspective, a smart CBA tool finds relevance in—and makes a novel contribution to—the emerging paradigm of data science and Big Data analytics in policy decision making.

Data analytics and Big Data have only recently begun to find applications in a policy decision making context, although this picture is rapidly changing (González-Bailón 2013) and such developments have also attracted critical inquiry (Gillingham and Graham 2017). Notwithstanding, the nascent field of ‘policy informatics’ (Johnston 2015), suggests that policy decisions can, and perhaps *should*, adopt an evidence-based and data-driven approach that draws on analytics techniques and the wealth of administrative records, open government data and user-generated data that are increasingly available. Rather than simply using such techniques and datasets to modify and/or enhance existing service delivery, data analytics and related methods reconfigure the nature of policy making itself (Henman 2010). Viewed in terms of the traditional policy cycle, a data science approach enables continuous evaluation. As Höchtl et al. (2016) propose, embedding data analytics into the policy cycle means that policy evaluation does not occur only at the end of the process, but continuously and in a manner that is transparent to stakeholders (see Höchtl et al. 2016, pp. 162–163).

To date, CBA tools are deployed in the context of single interventions and are therefore largely siloed from the broader context and from similar interventions. To make informed decisions about a given issue or intervention, agencies set up new interventions and estimates are provided by the tool. However, current CBA tools are not capable of learning from previous interventions, making use of intervention-relevant open datasets, making suggestions to the user based on similar data, or validating the subjective opinions of the user against other similar interventions or related datasets. Moreover, the categories and variables that are used in individual interventions are largely defined on a case-by-case basis by users, rather than drawn from a database of established categories and standards. Indeed, it is not possible to evaluate and exploit similarities between jurisdictions unless intervention data are shared or integrated between agencies - which is costly, difficult to manage and often ethically and legally infeasible.

A lack of data integration between interventions potentially has a negative impact on transparency and accountability, as different departments and agencies might make different decisions for the same policy problem. In turn, this hampers efforts to develop ‘joined up’ government in the era of digital governance that seeks to reintegrate otherwise disconnected governmental departments and public agencies (Margetts and Dunleavy 2013). The Smart MCBT proposed in this paper specifically aims to reddress the shortcomings of existing tools, by reintegrating individual CBA projects using a database system that securely stores and de-identifies project data, and redeploys it using a range of ML and data science techniques. The question of what works and what doesn’t is respecified by the Smart tool as a data science approach, which not only serves to enhance CBA but also reconfigure the policy making process in the paradigm of open data and data analytics.

This paper has focussed almost exclusively on the role of economic considerations in the selection of crime reduction interventions, using a gated community prevention approach as illustration. However, as the EMMIE acronym indicates, decisions about programme implementation invlove many more considerations than simply CBA (College of Policing UK 2017; Johnson et al. 2015). EMMIE highlights the difficult choices that often need to be made about programme implementation, choices that depend on much more than financial considerations. In particular, mechanisms, moderators and implementation factors highlight just how context-specific interventions may be—what works in one circumstance may not work in another seemingly similar situation. Moreover, as Johnson et al. (2015) point out, the available research on crime reduction activities varies enormously in quality. Information about costs is especially difficult to find. It is very noticeable that in the systematic reviews presented in the College of Policing UK (2017) Crime Reduction Toolkit, the economic costs cell is frequently empty. Likewise, we acknowledge that the current paper presents little in the way of empirical economic data. Our ambition with this paper has been limited to suggesting a way forward that might help redress the current dearth of high quality economic data around crime reduction activities.

## Conclusion

There are considerable opportunities and challenges for CBA using advanced data analytics, and the Smart MCBT proposed in this paper represents a step forward. Although the Smart MCBT is currently under development, future work will test and evaluate the concepts and methodology set out in this paper. Moreover, while we have focussed on the use of the Smart MCBT in crime prevention, it can be used in any policy context, whether it be concerned with crime, health, education, business management and so on. The question about whether, and how, ML and data science can improve and modify policy decision making remains an empirical question. Future evaluation of Smart MCBT is not restricted to the accuracy of cost estimates, but also a range of factors that includes user satisfaction metrics, time spent for data entry, frequency of input errors and the utility of knowledge discovery features for end users. At a broader scale, the role and meaning of such tools in a policy and governance context requires further critical attention. Although such tools offer numerous benefits and enhancements to policy decision making under austerity, attention must also be given to how data science and ML techniques and methodologies reconfigure the policy making setting itself—including the impact on stakeholders.

## Abbreviations

CCBA-DB: Calculated Cost–Benefit Analysis Database
CBA: cost–benefit analysis
CEA: cost-effectiveness analysis
CFA: cost-feasibility analysis
CSA: cost-savings analysis
CUA: cost-utility analysis
DV: dependent variable
EA: economic analysis
EMMIE: effect, mechanisms, moderate, implementation and economic
IVs: independent variables
ML: machine learning
MAR: missing at random
MICE: Multiple Imputation by Chained Equations
MI: multivariate imputation
SVM: support vector machines
MCBT: The Manning Cost–Benefit Tool
WSIPP: Washington State Institute of Public Policy’s

## Footnotes

1 The Manning Cost–Benefit Tools (excel version) can be downloaded free from http://csrm.cass.anu.edu.au/research/projects/manning-cost-benefit-tool or http://whatworks.college.police.uk/Research/Pages/Cost_Benefit_Tool.aspx; an online demo app version can be accessed from https://dmm.anu.edu.au/Criminology-Demo/—here an email will need to be sent to the lead author of this paper to request a username and login.

2 Analogous estimation uses a similar past project to estimate the duration or cost of a current project.

3 This method adopts the optimism correction bias scale providing a correction percentage for the estimation of cost according to the confidence grade of cost data.

4 The proportion of costs of an item that would be deemed as intervention-specific. The fraction can be estimated based on the proportion of time spent (e.g. a police officer spending 3 out of 7 working hours a day on the intervention) or space of a capital arranged for the intervention (e.g. an office building shared among different teams and units).

5 “Transfer payments are benefits that are received by an individual through special entitlement programs and other (usually government) mechanisms which redistribute income from one group to another” (Frisman and Rosenheck 1996, p. 534).

6 A measure of the relative harm from a crime or set of crimes that assist in the estimation of returns on investment from a given intervention. Refer to Curtis-Ham and Walton (2017) for an example of the application of a harm index.
